# Intraprostatic PSMA PET/MRI parameters and clinical markers for ISUP prediction and postoperative biochemical recurrence in patients with primary prostate cancer

**DOI:** 10.1007/s00259-026-07839-w

**Published:** 2026-03-23

**Authors:** Holger Einspieler, Michael Nürnberger, Clemens P. Spielvogel, Lukas Nics, Pascal A. T. Baltzer, Nicolai Huebner, Gero Kramer, Shahrokh F. Shariat, Bernhard Grubmüller, Marcus Hacker, Sazan Rasul

**Affiliations:** 1 Department of Biomedical Imaging and Image-guided Therapy, Division of Nuclear Medicine, Medical University of Vienna, Währinger Gürtel 18-20, Vienna, 1090 Austria; 2 Department of Biomedical Imaging and Image-Guided Therapy, Division of General and Pediatric Radiology, Medical University of Vienna, Vienna, 1090 Austria; 3 Department of Urology, Comprehensive Cancer Center, Medical University of Vienna, Vienna, Austria; 4 Department of Urology, University of Texas Southwestern Medical Center, Dallas, USA; 5 Division of Urology, Department of Special Surgery, The University of Jordan, Amman, Jordan; 6 Department of Urology, Second Faculty of Medicine, Charles University, Prague, Czech Republic; 7 Department of Urology, Weill Cornell Medical College, New York, USA; 8 Karl Landsteiner Institute of Urology and Andrology, Vienna, Austria; 9 Department of Urology and Andrology, University Hospital Krems, Krems, Austria; 10 Karl Landsteiner University of Health Sciences, Krems, Austria

**Keywords:** Prostate cancer, PSMA PET, Risk stratification, Prostate specific antigen, Tumor imaging

## Abstract

**Purpose:**

PSMA PET/MRI pairs high sensitivity for PSMA-avid lesions with superior soft-tissue contrast and provides quantitative metrics that may predict clinical outcomes of prostate cancer (PCa). We investigated whether intraprostatic PET-derived metrics and PSA-based variables predict histopathological ISUP grade and biochemical recurrence (BCR) after radical prostatectomy (RPE).

**Methods:**

Men with primary PCa who underwent [⁶⁸Ga]PSMA PET/MRI before RPE were retrospectively analyzed. PET-derived tumor metrics, initial PSA (iPSA), PSA density (PSAD), and ISUP grade were assessed for association with tumor aggressiveness and BCR. Discriminatory performance was evaluated using ROC AUCs. Multivariable logistic regression retained the better-performing variable within correlated pairs (e.g., SUV_peak_ vs. SUV_max_). Kaplan–Meier and Cox models examined recurrence-free survival.

**Results:**

Among 101 men, all evaluated parameters differentiated low- (ISUP 1–2) from high-grade (ISUP 3–5) disease, with PET metrics showing the highest AUCs. In multivariable analysis, SUV_peak_ independently predicted high-grade disease (OR 1.3, 95% CI 1.1–1.5, *p* < 0.001). Follow-up was available for 71 patients (mean 3.2 ± 2.8 years); 36 (51%) developed BCR after 2.7 ± 2.6 years. For BCR prediction, all variables except SUV_max_ showed significant AUCs. In multivariable models, ISUP (OR 1.8, 95% CI 1.2–2.9, *p* = 0.01) and PSAD (OR 4.0, 95% CI 1.1–15.0, *p* = 0.039) remained significant. The combined model (ISUP + SUV_peak_ + PSAD) achieved an AUC of 0.788 (95% CI 0.678–0.898, *p* < 0.001). In Cox analysis, only high ISUP grade independently predicted time to BCR (HR 3.2, 95% CI 1.2–8.9, *p* = 0.02).

**Conclusion:**

Intraprostatic PET metrics, especially SUV_peak_, effectively assess tumor aggressiveness before RPE, while PSAD adds prognostic value for recurrence. Postoperative ISUP grade remains the strongest predictor of BCR. Combined imaging and PSA-based markers may improve risk stratification and warrant validation in prospective studies.

**Supplementary Information:**

The online version contains supplementary material available at 10.1007/s00259-026-07839-w.

## Introduction

In primary prostate cancer (PCa), therapeutic decisions are mainly based on established risk stratification systems and histopathological features, such as the International Society of Urological Pathology (ISUP) grade group. While ISUP grades 1–2 represent low-risk disease, grade 3 indicates unfavorable intermediate risk, and grades 4–5 correspond to high-risk, aggressive tumors with poorer outcomes.

Commonly, patients with localized disease are treated with either radical prostatectomy (RPE) or radiation therapy, whereas those with localized but less aggressive and low-risk tumor types might only be managed with active surveillance [1]. Despite these curative-intent strategies, a significant proportion of patients experience biochemical recurrence (BCR), defined as a rise in PSA above 0.2 ng/mL after RPE [2]. The occurrence of BCR is an early indicator of treatment failure and is associated with disease progression and long-term adverse outcomes [3]. Thus, the identification of reliable imaging and laboratory biomarkers to improve risk stratification and guide therapeutic decision-making is of major clinical relevance.

Prostate-specific membrane antigen (PSMA) positron emission tomography (PET) is a non-invasive imaging modality used for the detection of PSMA-positive PCa lesions. Generally, over 90% of PCa patients show clinically relevant PSMA uptake, making PSMA-targeted PET integral to routine care [4, 5]. It is mainly used for primary staging, detection of biochemical recurrence (BCR), assessment in castration-resistant disease, and evaluation for PSMA radioligand therapy [6]. PET/computed tomography (CT) has demonstrated higher diagnostic accuracy than conventional CT and bone scintigraphy imaging with lower radiation dose and high potential in the detection of small, metabolically active lesions [7]. Building on this, quantitative PET imaging metrics, including the standardized uptake value (SUV), provide a non-invasive measure linked to disease burden and progression [8].

In addition, compared with PET/CT, PET/magnetic resonance imaging (MRI) offers superior soft-tissue contrast [9–11]. The MRI imaging component not only improves local staging by delineating intraprostatic tumor extent, including extra-prostatic extension and seminal-vesicle invasion [12], but also provides other useful information, such as prostate volume measurements enabling calculation of PSA density (PSAD) [13]. PSAD serves as a valuable prognostic biomarker by normalizing serum PSA levels to gland volume, thereby improving discrimination between benign PSA elevations and those associated with malignant or clinically significant PCa [14].

This study aimed to evaluate PSAD together with quantitative intraprostatic PSMA PET parameters derived from PET/MRI in primary PCa and to determine their individual and combined value for predicting histopathological grade and BCR after RPE.

## Methods

### Study cohort

We retrospectively analyzed a total of 177 patients who received a PSMA PET/MRI in the Division of Nuclear Medicine at the Vienna General Hospital from May 2014 to July 2021, due to initial staging for biopsy-proven primary PCa, and who subsequently underwent radical prostatectomy. In total, 76 patients were excluded from analysis: 22 owing to missing PSA data, 20 because of technical image issues, 22 without documented surgery, and 12 with a history of PCa treatment (radical prostatectomy, radiotherapy, hormonal therapy, or chemotherapy).

Finally, 101 patients diagnosed with primary PCa were included in this analysis. This retrospective study was approved by the Ethics Committee of the Medical University of Vienna (EK: 1745/2021) and was conducted in accordance with the Declaration of Helsinki. Owing to its retrospective nature, the requirement for written informed consent for data collection and analysis was waived.

### PET/MRI examinations

Diagnostic [⁶⁸Ga]PSMA PET/MRIs with a contrast-enhanced multiparametric MRI protocol focused on the prostate/primary tumor were performed on an integrated 3.0-T system (Biograph mMR, Siemens), as described in a previously published study [15]. Accordingly, patients received intravenous furosemide (20 mg) and hyoscine butylbromide (20 mg) immediately before [⁶⁸Ga]PSMA administration, and a urinary catheter was placed in all patients to optimize pelvic/abdominal image quality. Immediately after intravenous [⁶⁸Ga]PSMA-HBED-CC (2 MBq/kg), a 45-minute dynamic pelvic PET list-mode acquisition was started, followed by a partial-body PET from skull base to mid-thigh (four bed positions, 4 min each, sinogram mode). PET data were reconstructed with OSEM (3 iterations, 21 subsets); the last 10 min of the pelvic dynamic were summed for visual and semiquantitative analysis. MR-based attenuation correction used Dixon VIBE (in-/opposed-phase and fat-/water-only images).

### Image analysis and data collection

The PET/MRI images were analyzed using a dedicated workstation with Hybrid 3D software (version 4.17, Hermes Medical Solutions, Stockholm, Sweden). PET intensities, expressed in Bq/mL, were transformed into standardized uptake values (SUV). Two experienced nuclear medicine physicians extracted SUV_peak_ and SUV_max_ from the intraprostatic index lesions. Index lesions were defined as those with the highest Gleason score on the post-RPE histopathology report and were delineated on PET/MRI at the corresponding intraprostatic locations. Moreover, Prostate volume was measured on axial and sagittal T2-weighted MR images of the whole pelvis and PSA density (PSAD) was calculated from MRI-derived prostate volume and serum PSA.

Laboratory parameters at the time of PET/MRI, such as PSA, testosterone, carcinoembryonic antigen (CEA), and neuron-specific enolase (NSE) and clinical parameters (age, weight, height, and radical prostatectomy histology) were retrieved from the hospital information management system (AKIM). Follow-up data, including the date of the last PSA measurement and BCR status, were also recorded.

### Statistical analysis

Statistical analysis was performed using IBM Mac SPSS Version 30. Descriptive variables were demonstrated as absolute numbers (n), as mean ± standard deviation (SD), or as median (minimum; maximum). Shapiro-Wilk test and the Levene test were used for assessment and calculation of normality and heteroscedasticity of continuous data. Group comparisons between patients with and without BCR were performed using the Mann-Whitney U test. Spearman’s rank and Pearson’s correlation coefficients were used to assess associations between continuous and ordinal variables, depending on the distribution. Univariate discrimination was assessed with receiver-operating characteristic (ROC) analysis and the area under the ROC curve (AUC, 95% CI). For multivariable analysis, binary logistic regression evaluated associations of several variables. Right-skewed variables were log10-transformed prior to regression analyses to improve linearity with the logit, while approximately symmetric variables were analyzed on their raw scale. To limit collinearity, only the better-performing metric from each correlated pair (SUV_peak_ vs. SUV_max_; PSAD vs. PSA), as determined by univariate AUC, was entered into multivariable models. Model discrimination was quantified by computing AUC (95% CI) from the models’ predicted probabilities. Time-to-BCR was analyzed using Cox proportional hazards regression; odds ratios (OR) and hazard ratios (HR) are reported with 95% confidence intervals (CI). Kaplan–Meier curves (log-rank test) described BCR-free survival. As prediction of BCR is a patient-level outcome, all analyses were performed on a patient basis using the intraprostatic index lesion. Scatterplots and boxplots were used for visualization. Two-sided *p* < 0.05 was considered statistically significant. All p-values are to be interpreted exploratorily. A large language model (ChatGPT, OpenAI) was used to improve language and readability; all content was reviewed by the authors, who take full responsibility.

## Results

### Study cohort

Clinical and demographic characteristics of the studied 101 patients with primary PCa prior RPE are shown in Table 1. Overall, 63 patients had histologically confirmed ISUP grade 3–5 disease, while 38 patients had ISUP grade 1–2.

**Table 1:** Baseline clinical and demographic characteristics of the patients studied

Clinical parameters	Study cohort
Patients - n	101
Age in years – mean (± SD)	65.8 (± 7.4)
BMI - mean (± SD)	26.3 (± 3.4)
Blood parameter – Median (IQR)	
- iPSA ng/mL	11.19 (91.6)
- Testosterone	1.7 (3.7)
- CEA	2.6 (3.2)
- NSE	14.6 (11.4)
Prostate volume (mL) - Median (IQR)	26.7 (10.4)
PSA density - Median (IQR)	0.47 (2.0)
ISUP	
1	4
2	34
3	19
4	15
5	29
Intraprostatic PSMA lesion uptake - Median	
(IQR)	
SUV_peak_	10.3 (6.0)
SUV_max_	13.4 (6.8)

n: number; SD: standard deviation; BMI: body mass index; iPSA: initial prostate specific antigen; %: percentage; CEA: carcinoembryonic antigen; NSE: neuron-specific enolase; IQR: inter-quartile range; ISUP: International Society of Urological Pathology; SUV: standardized uptake value

### Correlation analyses between intraprostatic tumor PSMA uptake, PSA, PSAD and ISUP grade groups

While strong correlation was observed between iPSA and PSAD (*r* = 0.89, *p* < 0.001), several moderate correlations (*r* = 0.42–0.49; all *p* < 0.001) with statistical significance were found between SUV_peak_ and PSAD, SUV_max_ and PSAD, SUV_peak_ and ISUP (Supplementary Materials, Figure S1), SUV_max_ and ISUP, SUV_peak_ and PSA, and SUV_max_ and PSA. Weak, but still significant correlations were also determined between PSAD and ISUP, and PSA and ISUP (both *r* = 0.24; *p* < 0.05).

**Fig. 1:**
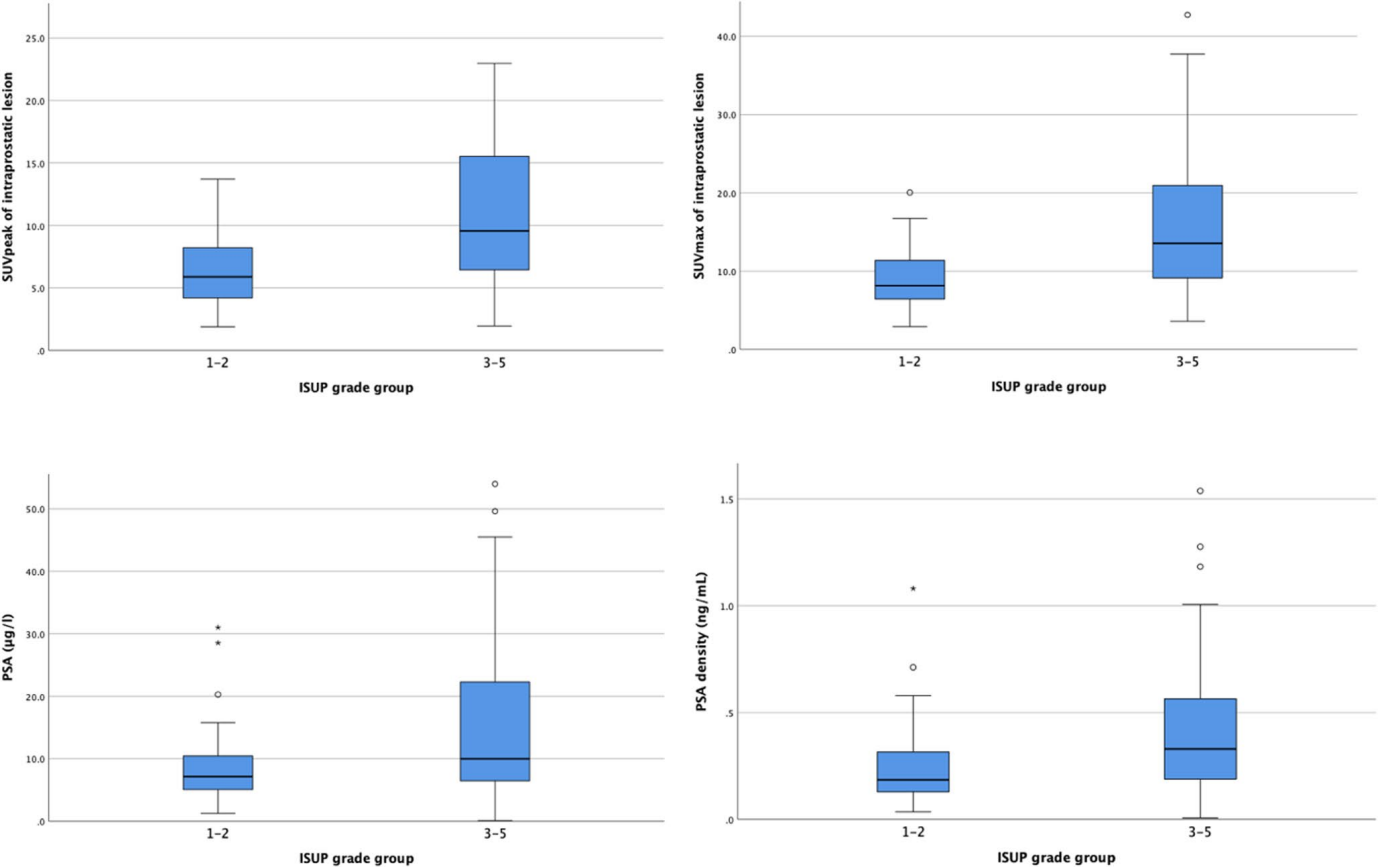
Boxplots of PSMA uptake values, initial PSA and PSAD in patients with low- and high-grade ISUP groups

In patients with an ISUP grade group ≥ 3, we found significantly higher SUV_peak_, SUV_max_, PSAD and iPSA values compared to patients with low-grade PCa (all *p* < 0.05), demonstrated in Fig. 1.

### Prediction of low- vs. high ISUP grade groups

All examined parameters (SUV_peak_, SUV_max_, PSA, and PSAD) demonstrated predictive ability for differentiating low- versus high-grade ISUP groups, with PET-derived parameters showing the highest discriminatory performance (Table 2).

**Table 2:** ROC analyses of imaging parameters predicting low vs. high ISUP grade groups

Outcome predicted	Predictor	AUC (95% CI)	*p*-value	Cut-off	Sensitivity	Specificity
**ISUP**	SUV_max_	0.753 (0.659–0.846)	< 0.001	12.9	58.7	86.8
	SUV_peak_	0.760 (0.667–0.0852)	< 0.001	8.5	64.5	84.2
	PSA	0.633 (0.525–0.742)	0.016	14.2	38.1	86.8
	PSA density	0.659 (0.550–0.767)	0.004	0.24	66.7	68.4

ROC: receiver operating characteristic; ISUP: International Society of Urological Pathology; AUC: area under the curve; %: percent; CI: confidence interval; PSA: prostate specific antigen; SUV: standardized uptake value

Among these parameters, PSAD demonstrated better discriminatory performance for ISUP grade prediction than PSA. Similarly, SUV_peak_ showed a higher AUC compared to SUV_max_. Therefore, PSAD and SUV_peak_ were included in the subsequent multivariable logistic regression models. In this analysis, SUV_peak_ (OR 1.3, 95% CI 1.1–1.5, *p* < 0.001) remained an independent predictor of ISUP, in contrast to PSAD (OR 1.1, 95% CI 0.4–3.1, *p* = 0.902). The combined model yielded an AUC of 0.759 (95% CI 0.667–0.852).

### Follow-up analysis of BCR

For the BCR analysis, only patients without evidence of metastatic disease on their pre-operative PET and with available follow-up data were included. Follow-up information was available for 71 patients, with a mean duration of 3.2 ± 2.8 years. BCR was observed in 36 patients after a mean of 2.7 ± 2.6 years. Among these patients, radiotherapy of the prostatic bed and/or metastatic lesions was administered in 28, hormonal therapy in 19, and chemotherapy in 6, following disease progression.

**Fig. 2:**
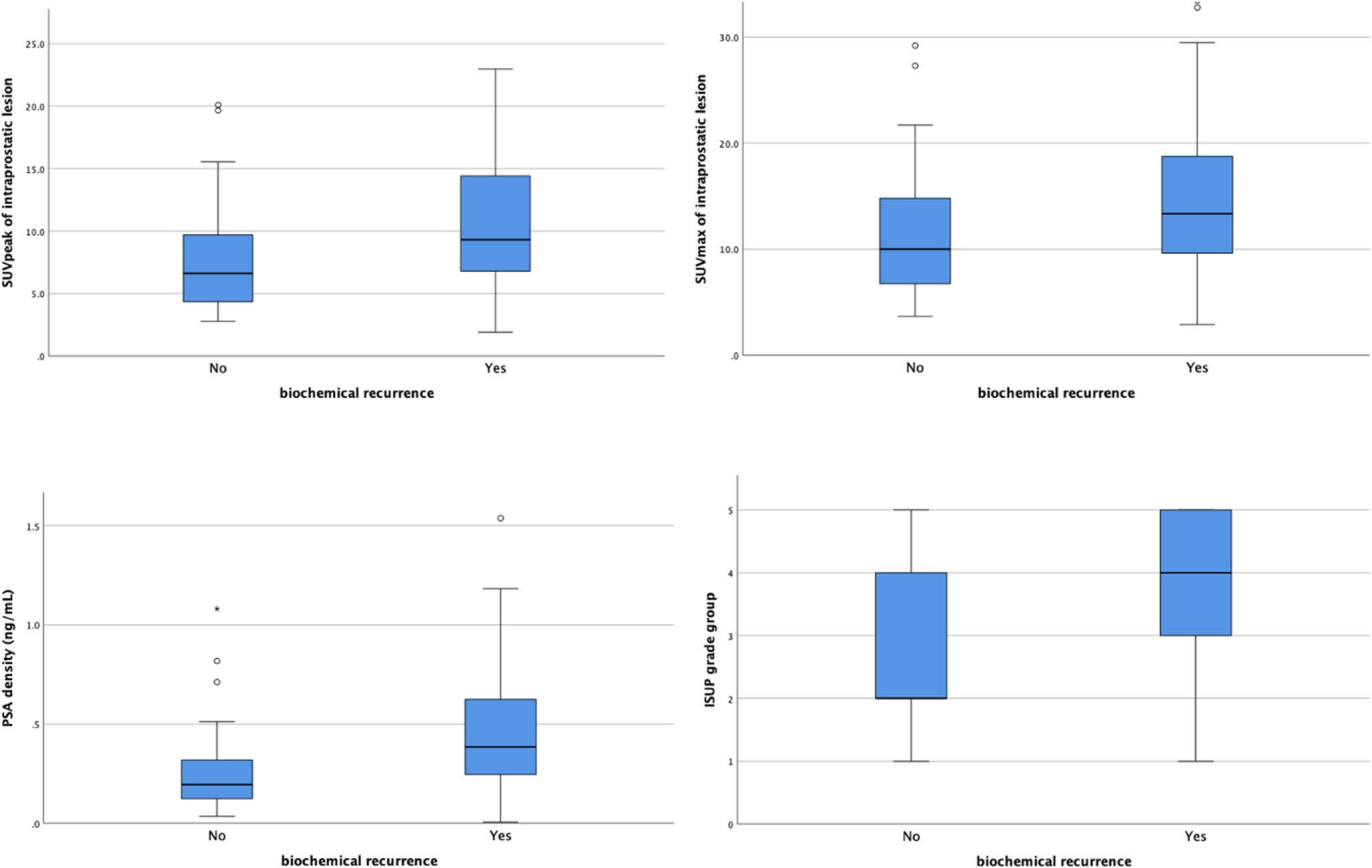
Boxplots of PSMA uptake values, PSAD and ISUP grade groups in patients with and without biochemical recurrence

Initial PSAD, iPSA, SUV_peak_, and ISUP were all significantly higher in patients who developed BCR compared with those who did not (all *p* < 0.05), Table 3; Fig. 2.

**Table 3:** Initial PSA values, PSAD, intraprostatic tumor PET/MRI metrics and postoperative ISUP of patients with and without BCR

Clinical parameters	No BCR (*n* = 35)	BCR (*n* = 36)	*p*-value
PSA - Median (IQR)	7.2 (8.9)	11.2 (17.0)	0.024*
PSA density - Median (IQR)	0.20 (0.2)	0.39 (0.39)	0.002*
SUV_peak_ - Median (IQR)	6.61 (5.4)	9.31 (7.8)	0.040*
SUV_max_ - Median (IQR)	10.01 (7.8)	13.35 (10.3)	0.154
ISUP- Median (IQR)	2.0 (2.0)	4.0 (2.0)	0.002*

n: number of patients; BCR: biochemical recurrence; PSA: prostate specific antigen; IQR: inter-quartile range; SUV: standardized uptake value; ISUP: International Society of Urological Pathology; *: statistically significant

### Prediction of time-to-BCR

In univariable Cox regression, PSAD (HR 2.5, 95% CI: 1.2–5.3, *p* = 0.02), iPSA (HR 1.0, 95% CI: 1.0–1.0, *p* = 0.008), SUV_peak_ (HR 1.1, 95% CI: 1.0-1.1, *p* = 0.041), and ISUP grade group (low vs. high) (HR 3.7, 95% CI: 1.4–9.6, *p* = 0.007) were each significantly associated with shorter time-to-BCR, while SUV_max_ (HR 1.0, 95% CI: 1.0-1.1, *p* = 0.052) showed a non-significant trend. However, in the multivariable model including PSAD, SUV_peak_ and ISUP (low vs. high), only ISUP remained independently predictive of time-to-BCR (HR 3.2, 95% CI 1.2–8.9, *p* = 0.025). Detailed results for the occurrence of BCR (binary prediction) are provided in the Supplementary Materials (Table S1).

## Discussion

Risk stratification in primary PCa relies on multiple clinical and imaging variables. The aim of this study was to evaluate intraprostatic PET/MRI-derived metrics such as SUV_peak_, SUV_max_, and PSAD alongside PSA, to compare their predictive performance and define their optimal use. When examined individually, all tested markers showed discriminatory performance for both histopathology and BCR in our study. Each parameter captures a different facet of disease biology: PSA is not specific to PCa and may be elevated in various prostatic conditions; in PCa it reflects basal-cell layer disruption and often correlates with disease burden [16]. PSAD additionally adjusts PSA for gland volume, whereas SUV metrics primarily reflect PSMA expression in tumor lesions [17]. Although some overlap is expected because higher tumor burden/aggressiveness can increase both PSA/PSAD and PSMA PET uptake, their independent performance supports complementary strengths. When analyzed together, however, subtle differences emerged.

In fact, quantitative PET metrics are increasingly used to predict histopathology, as can be seen in these previous reports [18–20]. While all these studies were performed with PET/CT, PET/MRI-derived SUV metrics show only minor differences and correlate strongly with those of PET/CT, especially in tumor lesions [21].

Although PSA, PSAD, tumor values of SUV_peak_ and SUV_max_ were significant predictors of low vs. high ISUP grade groups in our study, PET-derived metrics provided the greatest discrimination and remained the only independent predictor in multivariable models. Consistent with prior reports, SUV_max_ correlated moderately with ISUP, with a comparable ROC-derived cutoff around 12.9 [22, 23]. Interestingly, SUV_peak_ performed slightly better compared to SUV_max_. This likely reflects SUV_peak_’s greater robustness to image noise and reconstruction variability, as it averages uptake within a small volume around the hottest region rather than relying on a single voxel [24]. Consequently, SUV_peak_ provides more repeatable and biologically representative measurements. These findings indicate that PSMA uptake captures tumor aggressiveness best, while PSA and PSAD only add little value once PET intensity is considered.

For BCR prediction, ISUP, iPSA, PSAD, and SUV_peak_ were each significant in univariable analyses, whereas SUV_max_ showed a nonsignificant trend. However, after multivariable adjustment, only ISUP and PSAD remained independently associated with BCR, while PET uptake metrics no longer contributed independent information. Therefore, our study is in accordance with other studies, demonstrating that increased pretreatment PSAD and high-risk ISUP grade groups are independent indicators of BCR risk after initial treatment [25–27].

Clinically, our findings suggest that non-invasive imaging and laboratory markers may aid preoperative grading and risk stratification; however, PET-derived metrics from intraprostatic lesions are best interpreted as supportive rather than decisive on their own, given the modest sensitivity and specificity observed at the evaluated cut-offs. Yet, after surgery, histopathological assessment continues to represent the most dependable measure for predicting both the incidence and timing of recurrence. Taken together, these results suggest that while all parameters hold diagnostic value, their utility is context-dependent, varying between preoperative risk assessment and postoperative outcome prediction.

It must be emphasized that the present study has several limitations. First, its retrospective design and relatively small cohort size may limit the generalizability of the results. Second, the number of patients available for the BCR analyses was reduced due to incomplete follow-up data. Third, although MRI provides higher accuracy than other available methods, such as transrectal ultrasound, manual measurement of prostate volume may still introduce minor variations depending on the physician performing the assessment. Fourth, patients included in this analysis underwent [^68^Ga]PSMA PET/MRI for initial staging of primary PCa prior to surgery and were therefore enriched for high-risk disease, resulting in a low proportion of ISUP grade 1 and a relatively high proportion of ISUP grade ≥ 3 tumors, which may limit generalizability. Though these were outside the study’s scope, it is important to stress that we analyzed only intraprostatic PET-derived metrics and PSA-based variables; extraprostatic PSMA uptake, the PSMA PET PRIMARY score [28] and MRI parameters such as PI-RADS and signs of extraprostatic extension were not assessed, which might partly explain the stronger predictive performance of histopathology.

## Conclusion

In this study, all evaluated parameters, including PET-derived intraprostatic metrics, PSA, PSAD, and ISUP, provided meaningful predictive information for histopathological grade and/or BCR of PCa. Preoperative intraprostatic PET scan metrics were particularly informative for grading and risk stratification of primary PCa, whereas PSAD offered greater utility for predicting the occurrence of BCR. In the current analysis, nevertheless, the postoperative histopathology remained the most robust predictor of both the occurrence and timing of BCR. Given the modest sample size, these results should be interpreted cautiously; larger prospective, ideally multicenter, studies incorporating a broader range of clinical, imaging, and molecular variables are warranted to identify and validate the most informative predictors.

## Supplementary Information

Below is the link to the electronic supplementary material.

Supplementary Material 1

## Data Availability

The datasets generated and/or analyzed in this study are available from the corresponding author upon reasonable request.
