# Supplementary material for: Intraprostatic PSMA PET/MRI parameters and clinical markers for ISUP prediction and postoperative biochemical recurrence in patients with primary prostate cancer
Source: Eur J Nucl Med Mol Imaging. 2026 Mar 23;53(8):4905–12. doi: 10.1007/s00259-026-07839-w (PMC13249655; doi:10.1007/s00259-026-07839-w)
Supplement: Supplementary file 1 — Supplementary Material 1 [file 259_2026_7839_MOESM1_ESM.docx]

**Supplementary material**

### ****Figure S1.** Boxplots of intraprostatic PSMA uptake (SUVpeak) across ISUP grades**

### *
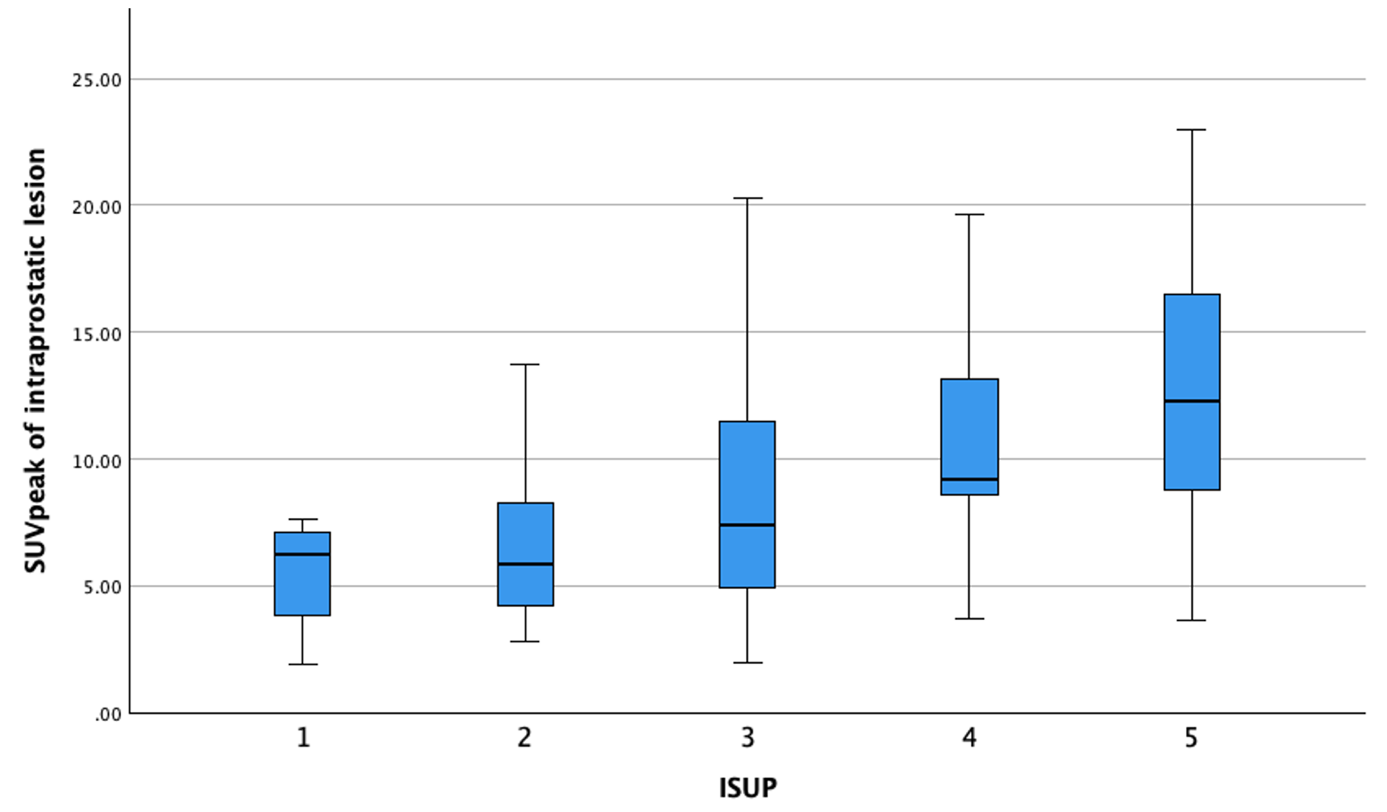
*

### ***Prediction of BCR occurrence***

### **All predictors achieved statistically significant AUCs for the prediction of BCR occurrence, except for SUV_max_, as can be seen in **Table S1**.**

### In the univariate ROC analysis for BCR, PSAD demonstrated superior performance compared to PSA, and SUV_peak_ achieved a higher AUC than SUV_max_. In addition, ISUP grade was a significant predictor of BCR. Therefore, PSAD, SUV_peak_, and ISUP were selected for multivariable logistic regression analysis.

### In multivariable logistic regression, ISUP (1-5) (OR 1.8, 95% CI 1.2–2.9, p=0.01) and PSAD (OR 4.0, 95% CI 1.1–15.0, p=0.039) remained independent predictors of BCR. No independent association was observed for SUV_peak_ (OR 1.0, 95% CI 0.9–1.2, p=0.5). The combined model yielded an AUC of .788 (95% CI 0.678–0.898, p<0.001).

**Table S1***.* ROC analysis of imaging and histopathological parameters predicting the BCR occurrence

| Outcome predicted | | Predictor | AUC (95% CI) | p-value | Cut-off | Sensitivity | Specificity |
| --- | --- | --- | --- | --- | --- | --- | --- |
| **BCR** | | SUV_max_ | 0.614  (0.495-0.732) | 0.059 | 10.5 | 75.0 | 56.9 |
|  | | SUV_peak_ | 0.651  (0.533-0.769) | 0.012 | 8.6 | 66.7 | 70.3 |
|  | | PSA | 0.663  (0.552-0.774) | 0.004 | 7.4 | 77.8 | 53.8 |
|  | | PSA density | 0.709  (0.602-0.817) | <0.001 | 0.28 | 72.2 | 67.7 |
|  | ISUP  (1-5) | | 0.702  (0.597-0.808) | <0.001 | 3.5 | 69.4 | 70.8 |

ROC: receiver operating characteristic.; BCR: biochemical recurrence; SUV: standardized uptake value; AUC: area under the curve; %: percent; CI: confidence interval; PSA: prostate specific antigen; ISUP: International Society of Urological Pathology
